# Perspectives of Pregnant and Breastfeeding Women on Participating in Longitudinal Mother-Baby Studies Involving Electronic Health Records: Qualitative Study

**DOI:** 10.2196/23842

**Published:** 2021-03-05

**Authors:** Austen Hentschel, Chu J Hsiao, Lynn Y Chen, Lauren Wright, Jennifer Shaw, Xinsong Du, Elizabeth Flood-Grady, Christopher A Harle, Callie F Reeder, Magda Francois, Adetola Louis-Jacques, Elizabeth Shenkman, Janice L Krieger, Dominick J Lemas

**Affiliations:** 1 Department of Health Outcomes & Biomedical Informatics College of Medicine University of Florida Gainesville, FL United States; 2 Department of Anthropology College of Liberal Arts and Sciences University of Florida Gainesville, FL United States; 3 Southcentral Foundation Anchorage, AK United States; 4 Clinical Translational Science Institute University of Florida Gainesville, FL United States; 5 STEM Translational Communication Center College of Journalism and Communications University of Florida Gainesville, FL United States; 6 Department of Obstetrics and Gynecology College of Medicine University of Florida Gainesville, FL United States; 7 Department of Obstetrics and Gynecology University of South Florida Morsani College of Medicine Tampa, FL United States

**Keywords:** electronic health records, pregnancy, breastfeeding, maternal-child health, research engagement, mother-infant medical record linkage

## Abstract

**Background:**

Electronic health records (EHRs) hold great potential for longitudinal mother-baby studies, ranging from assessing study feasibility to facilitating patient recruitment to streamlining study visits and data collection. Existing studies on the perspectives of pregnant and breastfeeding women on EHR use have been limited to the use of EHRs to engage in health care rather than to participate in research.

**Objective:**

The aim of this study is to explore the perspectives of pregnant and breastfeeding women on releasing their own and their infants’ EHR data for longitudinal research to identify factors affecting their willingness to participate in research.

**Methods:**

We conducted semistructured interviews with pregnant or breastfeeding women from Alachua County, Florida. Participants were asked about their familiarity with EHRs and EHR patient portals, their comfort with releasing maternal and infant EHR data to researchers, the length of time of the data release, and whether individual research test results should be included in the EHR. The interviews were transcribed verbatim. Transcripts were organized and coded using the NVivo 12 software (QSR International), and coded data were thematically analyzed using constant comparison.

**Results:**

Participants included 29 pregnant or breastfeeding women aged between 22 and 39 years. More than half of the sample had at least an associate degree or higher. Nearly all participants (27/29, 93%) were familiar with EHRs and had experience accessing an EHR patient portal. Less than half of the participants (12/29, 41%) were willing to make EHR data available to researchers for the duration of a study or longer. Participants’ concerns about sharing EHRs for research purposes emerged in 3 thematic domains: privacy and confidentiality, transparency by the research team, and surrogate decision-making on behalf of infants. The potential release of sensitive or stigmatizing information, such as mental or sexual health history, was considered in the decisions to release EHRs. Some participants viewed the simultaneous use of their EHRs for both health care and research as potentially beneficial, whereas others expressed concerns about mixing their health care with research.

**Conclusions:**

This exploratory study indicates that pregnant and breastfeeding women may be willing to release EHR data to researchers if researchers adequately address their concerns regarding the study design, communication, and data management. Pregnant and breastfeeding women should be included in EHR-based research as long as researchers are prepared to address their concerns.

## Introduction

Attempts to protect pregnant women by labeling them as a vulnerable population have played a role in excluding women, pregnant or not, from clinical research [1]. The difficulties in recruiting pregnant women for clinical trials are well documented [2,3], and tools such as the electronic health record (EHR) hold great potential for longitudinal mother-baby studies, ranging from assessing study feasibility to facilitating patient recruitment to streamlining study visits and data collection to providing data for retrospective observational studies. Longitudinal mother-baby studies are defined as studies that monitor the mother-baby pair beginning in pregnancy through the child’s first few years of life. Although the benefits to researchers of using EHR data are well discussed in the literature [4,5], the perspectives of the participants, who are key stakeholders in the clinical research process, are understudied. Understanding the perspectives of pregnant and breastfeeding women in EHR-based research is an important step toward engaging this population in future research studies.

Only a few studies have examined patients’ perspectives on the use of EHRs for research. Earlier studies suggested that less than a quarter of patients were willing to share their health records with researchers and even fewer were willing to share when their records contained sensitive information, such as HIV test results [6]. Later studies, likely corresponding with the increasing prevalence of the EHR, found greater willingness of study participants (ranging from 67% to 96%) to share with researchers [7-9]. Patients showed a strong preference for controlling which data would be available to whom [10-12] and were more likely to share deidentified data [6]. Trust in researchers was the strongest determinant of the level of protection desired for medical records and less trust correlated with a stronger desire for a more stringent EHR release process [11]. Patients also expressed concerns about the possibility that their data would fall into the hands of third parties, such as government agencies [6], for-profit organizations [10], and private health insurance companies [6]. Privacy, security, and trust in the research team were factors that impacted the decision to release the EHR to researchers.

Of the few studies to date that have explored the use of EHRs of pregnant women, most have only focused on the adoption of and engagement with EHRs through a patient-friendly portal that allows people to access their personal health information, to message providers, and to schedule appointments with providers [13-15]. Even studies on EHR portal use have largely been conducted in nonpregnant populations, despite indications that pregnant women are interested in web-based access to EHRs [13]. Engaging more pregnant women in longitudinal EHR-based research can help improve the scientific understanding of the developmental origins of health and disease. Successfully engaging this population in clinical research will require an understanding of their perspectives and concerns related to participating in EHR-based mother-baby studies. Therefore, we conducted an exploratory descriptive study of pregnant and breastfeeding women’s perspectives on releasing their own and their infants’ EHR data for longitudinal research to identify factors affecting their willingness to participate in research.

## Methods

### Overview

This qualitative study used semistructured, individual interviews with pregnant and breastfeeding women to elicit views about consenting to have their EHRs used for research. The reason for sampling from this population was to understand the perspectives and concerns of people who would be eligible for longitudinal mother-baby studies that use EHRs. The eligibility criteria mirrored those of a larger ongoing longitudinal mother-baby clinical study on the impact of breastfeeding on the infant gut microbiome (NCT03036696).

Individuals were deemed eligible to participate if they were aged between 18 and 40 years and were either pregnant or breastfeeding an infant under 12 months of age. Our study did not include English language fluency as an eligibility criterion. Exclusion criteria included a history of any of the following: inadequate breast milk production, pre-eclampsia, preterm delivery, or substance abuse during pregnancy. Thus, our sample represented those who would be eligible to participate in a real clinical study using EHR data. Participants were recruited through fliers posted at hospitals, restaurants, and grocery stores that detailed the study and included contact information for the research coordinator (MF). Participants were screened for eligibility over the phone, and interviews were scheduled upon confirming the participant’s eligibility.

### Approach

Trained interviewers (EF and MF) conducted all interviews from September 2017 to December 2018 in private rooms or offices on campus. A semistructured interview guide (Textbox 1) was used to elicit participants’ views on research involving the EHR. Participants were asked about their familiarity with the EHR and experience using an EHR patient portal, for example, to communicate with their health care provider. Questions also explored participants’ views about giving researchers access to both their own and their infants’ EHRs, length of time of access, and the inclusion of research results in their EHRs. Each interview lasted between 30 and 60 minutes, and the participants received an incentive of US $15. All semistructured interviews were audio recorded and professionally transcribed verbatim (Datagain). Transcribed interviews were stored in REDCap, a secure, web-based database platform. This study was approved by the institutional review board of the University of Florida (IRB201601909). None of the researchers involved had any conflicts of interest.

**Semistructured interview guide questions.**

1. How familiar are you with electronic health records and electronic portals?
2. Do you interact with your doctor using the electronic portal?
3. What length of time would you feel comfortable with the research team being able to access your medical records as part of a research study?
4. What length of time would you feel comfortable with the research team being able to access your infant’s medical records as part of a research study?
5. Would you want your research results to be included in the electronic health records?

### Data Analysis

Transcripts were organized using NVivo 12 software (QSR International). Qualitative and quantitative methods were used to analyze the data. The sample size was determined by reaching thematic saturation [16]. An iterative, inductive approach to thematic analysis was used to examine the data. Two coders (AH and LC) first read all the transcripts line by line and then developed a codebook that reflects both a priori and emergent themes (Table 1). In the first stage of analysis, the 2 coders independently coded each of the transcripts for a priori themes. Frequent discussions to resolve discrepancies in code application occurred between coders until consensus was achieved and emergent themes were identified. The final coded data set was further organized within a spreadsheet for subsequent exploratory analysis. The reliability of findings was enhanced by using a constant comparative method [17] in which coders compared subsequent transcripts with previous transcripts to confirm consistency of themes across data.

**Table 1:** A priori and emergent themes.

Themes	Description	Example quote
Concerns about privacy and confidentiality (a priori)	Factors pertaining to limited access of EHR^a^ data, including limiting of information related to stigmatizing conditions, deidentification of records, and release without consent to third parties	“I would like to have control over as much of my privacy as I can.”
Role of transparency by the research team (a priori)	Factors related to full disclosure about the research being conducted, the purpose for which medical records are being used, and the need for researchers to reobtain consent from participants for future use of EHR data	“Yeah, I don’t know the answer. I guess it would have to be I would have to know a little bit more about what the study would be that would require my medical records before I’d say yes or no.”
Concerns about surrogate consent (emergent)	Parent or legal representative concerns about consenting for their neonate to participate in clinical research, including the length of access to the child’s record and how release of the child’s EHR could affect the child later on	“Yes, I guess. That’s a hard one for me to answer. Here’s why. It’s because I’m answering for a child who doesn’t have a say...”

^a^EHR: electronic health record.

## Results

### Participants

Participants included 29 women who were either breastfeeding (n=10) or pregnant (n=19). The demographic characteristics of the participants are presented in Table 2. The age range of participants was from 22 to 39 years, with most (66%) in their 30s. The education level varied from an associate degree to a professional degree, with most (83%) having a bachelor’s degree or higher. Most participants described their race as White. The racial characteristics of our sample were similar to those of a local county [18].

**Table 2:** Participant characteristics (N=29).

Characteristics		Values, n (%)
**Age group (years)**		
	20-29	10 (34)
	30-39	19 (66)
**Education**		
	Professional or graduate degree	17 (59)
	Bachelor’s degree	7 (24)
	Associate degree	3 (10)
	Tech or vocational degree	2 (7)
**Race or ethnicity**		
	Black	5 (17)
	White	20 (69)
	Other	1 (3)
	Missing	3 (10)
**Familiarity with EHR^a^ and EHR portals**		
	Familiar	27 (93)
	Not familiar	1 (3)
	Not asked	1 (3)
**Willingness to release own EHR**		
	Yes	18 (62)
	Ambivalent or conditional yes	11 (38)
**Willingness to release infant’s EHR**		
	Yes	18 (62)
	Ambivalent or conditional yes	7 (24)
	Missing	4 (14)
**Length of time of EHR release**		
	Equal or longer than the length of the study	12 (41)
	Others	12 (41)
	Missing	5 (17)

^a^EHR: electronic health record.

### Familiarity With the EHR

Almost all participants (27/29, 93%) had existing knowledge of and were familiar with the EHR and EHR portals. Participants were coded as being familiar with an EHR portal if they could provide specific examples of how they used it, such as communicating with a physician, checking in for appointments, or viewing test results. Most participants primarily used the EHR to update their health information and view the test results. Notably, 2 participants also had experience interacting with an EHR system for their jobs. Many participants used EHR portals to communicate with providers, including 3 participants who stated that they did this primarily during pregnancy and 1 who stated that it was her preferred method to ask questions because phone calls had a much longer follow-up period. One participant reported preferring to converse with providers in person.

### Willingness to Release Records

Most participants were willing to release their own and their infants’ EHRs to the researchers. Willingness to provide access to EHR data fell within 2 categories: full EHR release and conditional EHR release. Full EHR release was characterized by participants being completely comfortable and willing to release their own and their infant’s EHR for research purposes and without conditions. Conditional EHR release reflected ambivalence about sharing EHR data and was characterized by the participants’ willingness to provide restricted or stipulated access to their EHR (eg, “Researchers can access my data as long as they are transparent about its use”). Nearly half of the participants (n=12) were willing to make their own and their infants’ EHRs available to researchers for the duration of a research study or longer. More than one-third of the respondents (n=11) expressed conditional agreement about releasing their EHR for research. The salient themes of participants’ concerns regarding EHR release are described as follows.

### Salient Themes of Participant’s Concerns for Releasing EHRs

Although participants were familiar with EHR portals and willing to release their EHRs for research, they articulated several concerns. Concerns centered around 3 themes, including privacy and confidentiality, transparency by the research team, and surrogate consent for infants. Finally, we share patient insights into how the EHR portal may be used for research engagement.

#### Privacy and Confidentiality

Privacy and confidentiality concerns included whether information would be dispersed without prior consent; the types of personal information that would be used in the study, including access to stigmatized health information; and whether deidentification would be used. Participants were particularly concerned with anonymity and were interested in sharing both their own and their infants’ EHRs if the information was deidentified (Textbox 2).

**Quotations representing concerns about privacy and confidentiality when releasing electronic health records for research.**

- “That’s the only other thing that comes to mind is that maybe it would be deidentified and maybe not use her face along with that if that makes sense.” [BIS014]
- “They don’t need all of my medical records…I look at the big scale, just the internet today, and how everybody has access to everything, and how there’s crazy stuff politically and crazy people, if someone were to ever take advantage, I would like to have control over as much of my privacy as I can.” [BIS003A]
- “I’m sure there’s people with certain conditions like HIV and stuff like this who wouldn’t want that type of stuff to be exposed.” [BIS030]
- “I say, this should be like in a secured and it shouldn't be shared with others without permission.” [PRG003]
- “I have a very easygoing pregnancy, no complications...So, I’d be comfortable. I don’t know if another mom would be if they had some complications or genetic history or whatever.” [PRG016]

Participants needed assurance that the EHR data would be secure, with limits on who could access the data. In particular, participants were concerned about the possibility that their data might be shared with third-party institutions, such as health insurance companies:

My concern would be if in the research study, anything like if anything came back long term genetic...I don't want connected [to my EHR] because of getting health insurance. [If] I have to get my own plan, how pre-existing conditions will affect it...that would be my biggest concern. Just because I know I don't, I don't trust the state of health care in the country right now.

PRG010

Participants also raised concerns about giving researchers unlimited access to EHRs and providing access to stigmatizing health conditions in their EHRs. One breastfeeding woman stated that she was uncomfortable releasing provider notes that included stigmatizing or potentially embarrassing conditions:

I think the only way I would maybe not feel comfortable is if I had some sort of alcohol or substance use disorder, if I engaged in an activity that was embarrassing for me, things that are stigmatized, if I had mental health issues. I'm lucky I don't, so I don't have an issue, or if I had HIV or some other infection like that. Yeah, basically any stigmatizing conditions, I might not be open to allowing people to seeing my her.

BIS001

In addition to substance use disorders, participants were concerned about general mental health conditions, miscarriages, medical conditions unrelated to pregnancy, HIV, and genetic panels of their infants. People were less willing to share information about medical conditions that were perceived to be more stigmatized.

#### Transparency by the Research Team

Participants also discussed the importance of transparency by the research team in their decision to release their EHRs (Textbox 3). Transparency is described as full disclosure of the research being conducted and the purpose for which medical records are being used. Research team transparency also includes the need for researchers to reobtain consent from participants for future use of EHR data. Participants expressed fear regarding how the information in the released EHR would be used by the research team. They also wanted the study personnel to clearly explain the specific EHR elements (ie, data points) needed for the study and how the information would be used, with a justification for the length of time records to be accessed.

**Quotations representing the role of transparency by the research team when releasing electronic health records for research.**

- “It just depends on how they are going to use that data.” [BIS009]
- “I guess it would have to be I would have to know a little bit more about what the study would be that would require my medical records before I'd say yes or no.” [BIS013]
- “I guess, I would wanna know and understand why the research team would need continuous access...Throughout the study like what information do you need after like getting my blood type and, you know, my initial like assessment of where I’m at.” [PRG016]

One participant remarked on the complexity of conducting research and the possibility of needing to reconsent at a later time point in longitudinal studies:

If you’re studying developmentally how the child is changing and how good health is affecting that. A lot of times, some of these things aren’t diagnosed till later. But do you probe first the parent and then decide whether you're going to collect...I don’t know. I don’t know if this would just be an open thing where they can do it at any time, but it's like, we're monitoring and then we go, “We’re seeing a trend and we want to collect the data on the medical records and this information. Does the parent approve? This is why,” and explain to the parent how it could be helpful for future children type of thing.

BIS003

#### Surrogate Consent

Although many participants were comfortable releasing their own and their infant’s EHR for research purposes, others expressed uncertainty. In particular, participants were concerned with providing surrogate consent, which was described as a concern over hypothetical situations in which the child may later disagree with the parent’s decision to participate in the study. One participant shared:

How is this going to impact him when he's older?...You know, where does this information go? Could it ever potentially become something that's limiting or “Mom, why did you release my information to this,” you know? Like, “Why do these people keep contacting me? I don't want to participate.”

BIS011

In one instance, the participant provided a hypothetical example of how her surrogate decision making may intrude on her child’s autonomy in deciding who is privy to the child’s protected health information:

That’s a hard one for me to answer. Here’s why. It’s because I’m answering for a child who doesn’t have a say, and maybe they wouldn’t, one day, like that information out there. Especially if there’s some condition they may end up having later that we don’t know, like autism or whatever.

BIS003

This discomfort reflects concerns over unpredictable future consequences resulting from their surrogate consenting on behalf of their child to release their child’s EHR to researchers. These persons were keenly aware that their decisions may have a lasting, unforeseen impact on their children.

### Research Results in EHRs

Researchers can write research notes in an EHR, which become a part of a patient’s medical record. Laboratory tests ordered for research instead of clinical care may be included in the EHR. In the final part of the interview, participants were asked, if given the option, whether they would prefer their research results to be included in their EHRs. Participants were overwhelmingly interested in being able to access their research results (eg, laboratory test results conducted as part of a clinical research study) in their EHRs. One participant shared how receiving results would make her feel like she “is a part of a bigger picture...and doing something important” (PRG014). Furthermore, feedback from the research team in the form of research-generated results through the EHR was noted as a strategy to enhance transparency and improve trust in the research process:

Yeah, I think it’s a good thing because I can't see, so how they use my information, my reports and I’m aware of the process...if they can share some results with me, or at least tell me what they are doing with my records and information, it makes me more happy and confident about the process...And I can trust them.

PRG011

A few participants expressed ambivalence about receiving research-generated results through their EHRs. Participants did not want research results to be included if they revealed a stigmatizing condition, such as being a *heroin addict*. Others did not want their research results to be included as part of their permanent record, owing to concerns regarding the physician’s ability to interpret research results, which may complicate care.

## Discussion

### Principal Findings

The aim of this study was to understand the concerns and reservations of pregnant or breastfeeding women about participating in longitudinal mother-baby studies that use EHRs. The participants in our study were largely familiar with the EHR, many gaining familiarity through access to their own EHR. More than half of the pregnant and breastfeeding participants were willing to share their EHR data with the researchers. This finding is similar to that of the research on nonpregnant patients’ willingness to share their EHR data [8,9]. In our study, participants wanted to be informed about how researchers were using their EHR data and to retain control over which elements of the EHR were released. In a 2019 study, more than three-fourth of participants who were given a list of EHR data elements to share with researchers chose to withhold at least one item [9]. This control may be an important component of a person’s willingness to participate in a study.

### Integrating Research and Health Data

A salient topic discussed by participants was integrating research data with health data in the EHR. Several participants advocated the release and availability of research test results in the EHR. Previous studies support this finding that women overwhelmingly want to actively engage in their health care through the use of EHRs [13,19]. Furthermore, these studies found that pregnant women were significantly more likely to log in to the EHR portal when they could view their personal antenatal health record [20], and the majority of those who created an EHR portal account would use it again for future pregnancies [14]. Moreover, in recent years, organizations such as the National Institutes of Health and the National Academy of Sciences have increasingly demanded that individual research results be shared with participants in biomedical research. This accessibility creates a patient-centric approach to research, which provides a level of transparency that may increase both trust in the research team and future participation in research [21]. Thus, there is an exciting potential for EHRs to encourage research participation. Perhaps a research portal interface with the EHR can help researchers engage populations historically excluded from clinical research. The finding that accessibility to research test results was viewed as improving transparency and trust in the research team suggests that a research portal interface can also help repair the broken trust in research held by certain populations. The integration of health and research uses of the EHR may also be beneficial during a time when participants might have reservations about making additional in-person visits to the hospital, such as during a pandemic. However, using the EHR to encourage active research participation may also perpetuate disparities in research participation as race, education level, and internet access have been shown to affect EHR engagement [22].

### Versatility of the EHR

Some participants also recognized that the EHR is a 2-way street: not only are health data going to researchers but research data may also go to health care providers. The integration of research and health data within a health system also highlights the potential for research to help serve those in lower resource settings: research dollars could possibly pay for tests or laboratories that may otherwise be unavailable to patients. Research results also have the potential to serve as a point of health care intervention (eg, screening out a prospective participant because of abnormal results on a laboratory test can serve as an opportunity to refer a patient to an appropriate physician), although participant opinions ranged from doubt (inability of doctors to interpret research results) to objection (in the case of stigmatizing conditions). A frequently cited concern was that a stigmatized condition (eg, substance abuse) discovered during a research study may be shared with health care providers and other third-party vendors, such as health insurance companies. Although perceived stigma led to an emphasis on privacy and confidentiality, we also found that trust was important in mitigating these concerns. Trust in the research team to deliver promises of privacy and confidentiality was an important component of research participation and EHR release. Although all participants hypothetically spoke about having a stigmatized condition, their concerns reflected a real issue of selection bias in EHR research. Patients with stigmatized conditions may be less likely to opt for EHR research studies, which would affect the representativeness of EHR data and compromise generalizability.

### Limitations

Although thematic saturation was reached for pregnant or breastfeeding participants’ concerns about releasing their own and their infants’ EHRs for research, a limitation of our study is the lack of racial and educational diversity and may play a role in the themes identified in the results. For example, previous studies have found that Black participants and those reporting lower education were less trusting of medical researchers and spent more time during the consenting process [8]. Further investigations of racially and ethnically diverse obstetric patients’ familiarity with EHR-based research and their perspectives on releasing EHR data to researchers are warranted. Moreover, this study excluded people with previous pregnancy complications, which may have introduced selection bias. Future studies should explore whether this group exhibits different views on sharing EHR data for research. In addition, access to and familiarity with EHRs is predicated on having internet access, which indicates the need to include more educationally and socioeconomically diverse populations.

### Conclusions

Previous qualitative studies within the pregnant population have focused on understanding their perspectives on antibiotic use to develop tailored perinatal health education interventions to increase knowledge, particularly using EHRs, to provide additional information on antibiotic use [23]. This is the first qualitative study to explore the perspectives of pregnant or breastfeeding women on participating in EHR research and provides significant insights into their attitudes toward sharing their own and their infants’ EHRs. Participants were largely familiar with engagement of their EHR for health care purposes, and most of them were willing to release their EHRs to researchers, provided their concerns for privacy, confidentiality, and transparency were addressed. Participant responses suggested that the EHR may play an underappreciated role in clinical research by providing research-generated test results to participants. This finding marks a departure from a singular focus on only studying the use of the EHR for health engagement toward use for research engagement among pregnant and breastfeeding women. How the EHR can be mobilized to better engage populations traditionally excluded from clinical research is an important topic for future studies.

## Abbreviations

EHR: electronic health record
